# Supplementary material for: High order expression dependencies finely resolve cryptic states and subtypes in single cell data
Source: Mol Syst Biol. 2025 Jan 2;21(2):173–207. doi: 10.1038/s44320-024-00074-1 (PMC11790937; doi:10.1038/s44320-024-00074-1)
Supplement: Supplementary file 1 — Appendix [file 44320_2024_74_MOESM1_ESM.pdf]

Appendix for High order expression dependencies finely resolve cryptic states and subtypes in single cell data

Contents

Appendix for High order expression dependencies finely resolve cryptic states and subtypes in single cell data 1
A.1. Nextflow runtime on HPC cluster 1
A.2. Robustness to gene selection 1
A.3. Simulations 1
A.4. Comparison with multimodal data 5

A.1. **Nextflow runtime on HPC cluster.** Stator's nextflow pipeline is represented in Appendix Figure S3. Stator computes combinatorial 2-point up to 7-point gene interactions, if estimable. The complete set of interactions among 1,000 HVGs is not computationally tractable: half a million 2-point interaction (1,000 choose 2), 166 million 3-point interactions, 41 billion 4-point interactions, 8 trillion 5-point interactions and so on. Instead, Stator first uses the Peter-Clark constraint-based algorithm (Spirtes et al., 2001), in order to perform (conditional) independence tests in order of increasing complexity, starting from a fully connected (undirected) graph of genes. Once a primary skeleton is obtained via the PC algorithm, Stator then uses a score based MCMC algorithm to improve the accuracy of an estimated Directed Acyclic Graph (DAG), using the hybrid approach introduced by Kuipers et al. (2022). This massively reduces the search space for potentially significant interactions. Note that memory requirements and runtime depend on gene expression connectivity in the given tissue and condition of interest. The sparser the dependence structure, the lower are the memory and runtime requirements which are unknown prior to running Stator's nextflow pipeline. For a mouse embryonic brain dataset with 19,000 cells, the maximum memory requirement was 32GB for running the nextflow pipeline.

A.2. **Robustness to gene selection.** Stator's robustness to an increasing number of genes for identifying significant MFIs is investigated more fully elsewhere (Jansma (2023b) Chapter 4,"Robustness to gene selection"). More specifically, it is demonstrated that when increasing the number of HVGs from 300 to 700, all significant 1-point interactions, $> 98\%$ of 2-point interactions, and approximately 96% of 3-point interactions kept the same sign and 95% confidence interval. For completeness, we additionally show in Appendix Figure S5 that the number of genes in the Markov Blanket (MB) stabilises at ~ 15 for increasing numbers of HVG; the MB is the smallest set of genes G conditional on which Gene i and Gene j are independent of all other genes. This means that the number of MFI is also stable at higher HVG number because stochasticity only arises due to variation in MB genes.

A.3. **Simulations.** In previous work, the core MFI estimator of Stator, for quantification higher-order dependence amongst variables, was demonstrated to successfully recover ground truth results in extensive sets of simulation studies (Beentjes and Khamseh, 2020). We performed 2 further simulations using the RP dataset: (a) One in which a fictitious d-tuple of genes was induced and then detected by Stator and, (b) another in which a cell state (blood contamination) was removed from the Stator run and, indeed, was then not detected. Specifically, since we are interested in biological reproducibility, rather than technical reproducibility, we do not demand that

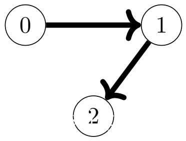

Chain							
Genes	Interaction	F	ρ_p	p-val	Partial ρ_p	p-val	MI
[0, 1]	4.281	0.000	0.790	0.0	0.635	0.000e+00	0.515
[0, 2]	0.056	0.117	0.622	0.0	0.031	2.261e-23	0.301
[1, 2]	4.249	0.000	0.786	0.0	0.628	0.000e+00	0.510
[0, 1, 2]	-0.052	0.217	NaN	NaN	NaN	NaN	0.300

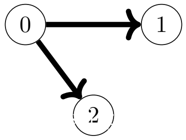

Fork							
Genes	Interaction	F	ρ_p	p-val	Partial ρ_p	p-val	MI
[0, 1]	4.268	0.000	0.789	0.0	0.634	0.000e+00	0.514
[0, 2]	4.257	0.000	0.788	0.0	0.632	0.000e+00	0.512
[1, 2]	-0.014	0.376	0.622	0.0	0.028	6.518e-19	0.300
[0, 1, 2]	0.020	0.376	NaN	NaN	NaN	NaN	0.300

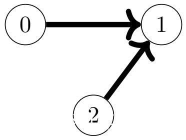

Additive collider							
Genes	Interaction	F	ρ_p	p-val	Partial ρ_p	p-val	MI
[0, 1]	2.144	0.000	0.395	0.000	0.505	0.000e+00	1.154e-01
[0, 2]	-0.989	0.000	-0.002	0.593	-0.070	5.172e-109	2.059e-06
[1, 2]	2.144	0.000	0.395	0.000	0.505	0.000e+00	1.154e-01
[0, 1, 2]	0.003	0.438	NaN	NaN	NaN	NaN	-2.678e-02

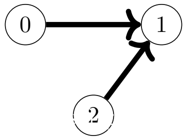

Multiplicative collider							
Genes	Interaction	F	ρ_p	p-val	Partial ρ_p	p-val	MI
[0, 1]	0.032	0.140	0.427	0.000	0.478	0.000e+00	1.403e-01
[0, 2]	-2.156	0.000	-0.005	0.145	-0.087	1.463e-166	1.529e-05
[1, 2]	0.036	0.109	0.429	0.000	0.480	0.000e+00	1.415e-01
[0, 1, 2]	4.237	0.000	NaN	NaN	NaN	NaN	-1.150e-01

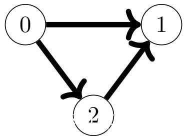

Additive collider + chain							
Genes	Interaction	F	ρ_p	p-val	Partial ρ_p	p-val	MI
[0, 1]	2.103	0.000	0.705	0.0	0.362	0.0	0.396
[0, 2]	3.288	0.000	0.790	0.0	0.599	0.0	0.515
[1, 2]	2.113	0.000	0.706	0.0	0.364	0.0	0.397
[0, 1, 2]	0.050	0.162	NaN	NaN	NaN	NaN	0.335

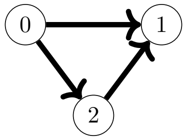

Multiplicative collider + chain							
Genes	Interaction	F	ρ_p	p-val	Partial ρ_p	p-val	MI
[0, 1]	-0.017	0.342	0.709	0.0	0.365	0.0	0.403
[0, 2]	2.094	0.000	0.786	0.0	0.596	0.0	0.510
[1, 2]	-0.057	0.092	0.707	0.0	0.361	0.0	0.401
[0, 1, 2]	4.359	0.000	NaN	NaN	NaN	NaN	0.293

Appendix Figure S1: Values used for Figure EV1. From each graph, we generated 100k samples from a Bernoulli distribution with $p = 0.5$ and added zero-mean Gaussian noise ($\sigma = 0.4$) before binarising. To quantify the significance value of the interactions, we generated 1,000 bootstrap resamples of the data, and calculated F: the fraction of resampled interactions that have a different sign from the original interaction. A smaller F corresponds to a more significant interaction.

a state is exactly reproduced in terms of its defining d-tuples, but rather that the list of genes that defines a state and their expression are sufficiently similar. For this, we refer to a gene together with its binary expression as a 'gene state'. Given a Stator state S from the original run, and a state T arising from a new data set, we say that S and T are similar if they share at least two gene states among the 30 most commonly occurring gene states in each state. If,

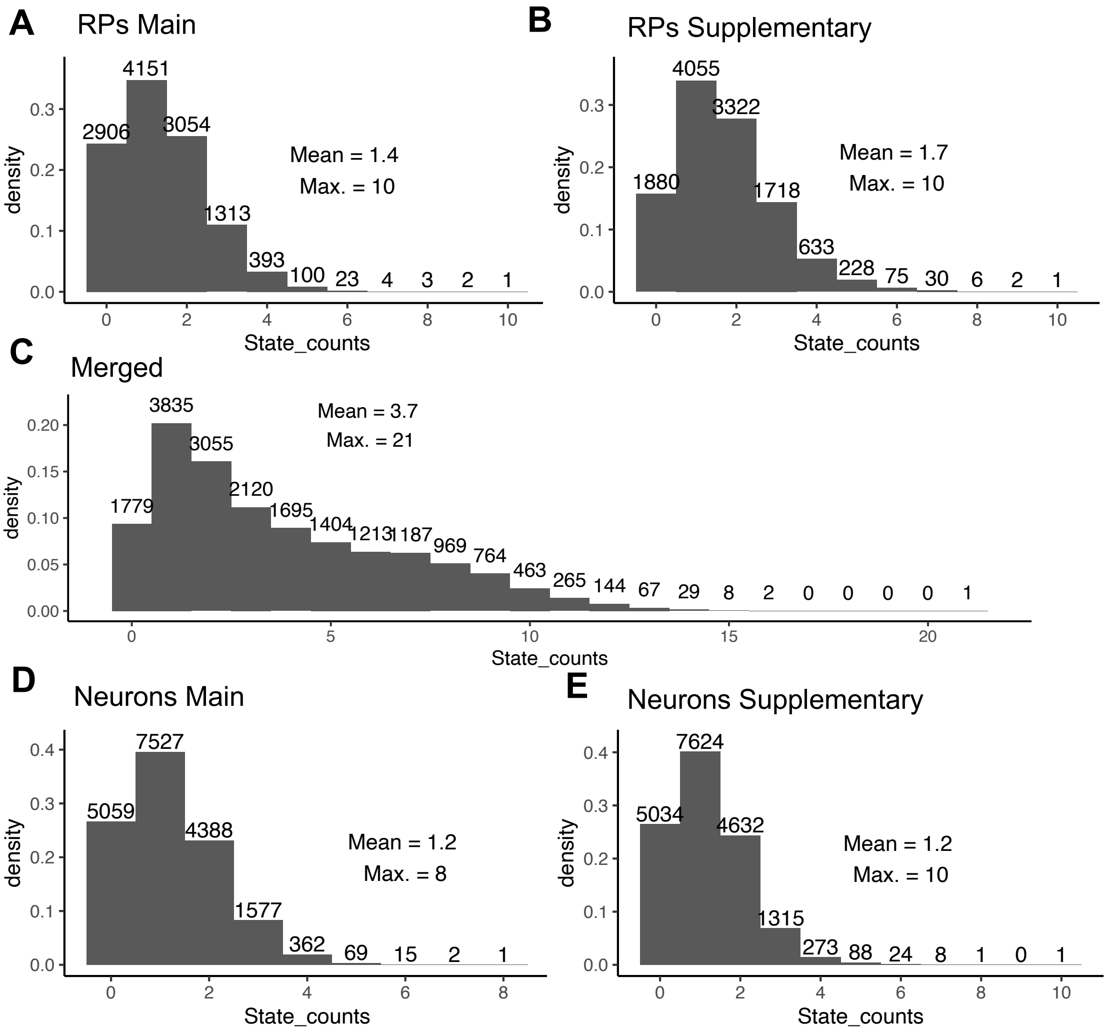

Appendix Figure S2: **Histograms of the number of cells labelled with variable numbers of states.** Results are for two disjoint embryonic radial glial cell-like cells (A and B), for the merged RP and neuron set (C), and for two disjoint sets of developmental neurons (D and E). Numbers shown above the bars indicate how the number of cells with the specific number of labels (X-axis).

however, the Stator state is made up of only three gene states, then sharing a single gene state is sufficient to be considered similar. For example, if S and T are both composed of two d-tuples: $S = ([A+, B+, C-], [A+, C-, D+])$ and $T = ([C-, E+, F-], [A+, G-, H+])$, then together they are composed of the eight gene states $(A+, B+, C-, D+, E+, F-, G-, H+)$, of which $A+$ and $C-$ are shared. Since S and T share at least two gene states, we conclude that they are similar. If, instead, $T = ([C-, E+, F-], [G-, H+, I+])$, then they are not similar. Finally, if $T = ([C-, E+, F-])$, then T is composed of just three gene states, so the single shared gene state $C-$ suffices to make S and T similar.

For simulation (a), a fictitious d-tuple of genes was induced by randomly choosing 90% of the cells where (Cited2, Basp1, Fhl1) $= (1, 0, 1)$ and changing this to $(1, 1, 1)$. Stator was then re-run on

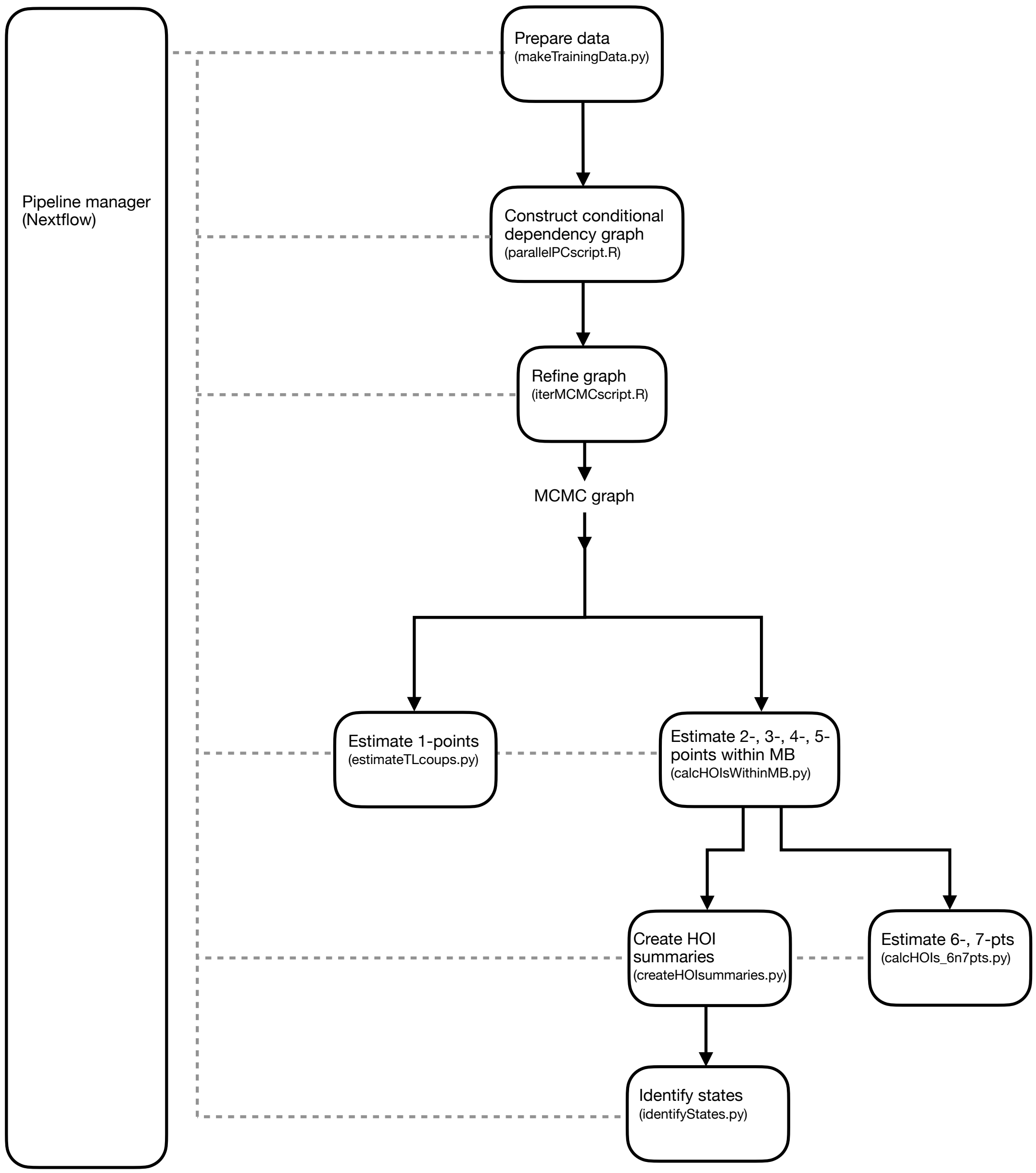

Appendix Figure S3: **Stator's nextflow pipeline.** From data preparation to groupings of d-tuples used for state identification. Abbreviations: HOI, Higher-order interactions; MB, Markov blanket.

the new data which included the original set of genes together the induced tuple. D-tuple (Cited2, Basp1, Fhl1) = $(1, 1, 1)$ was recovered as part of state 14 in Appendix Figure S7 (panel A) with FDR < 0.05 and > 6-fold enrichment, at the default Dice dissimilarity threshold. For simulation (b), the cells tagged as blood contamination in the original run were removed and Stator was re-run. The results in Appendix Figure S7 (panel B) indicate that the blood contamination state does not map to any states in the run.

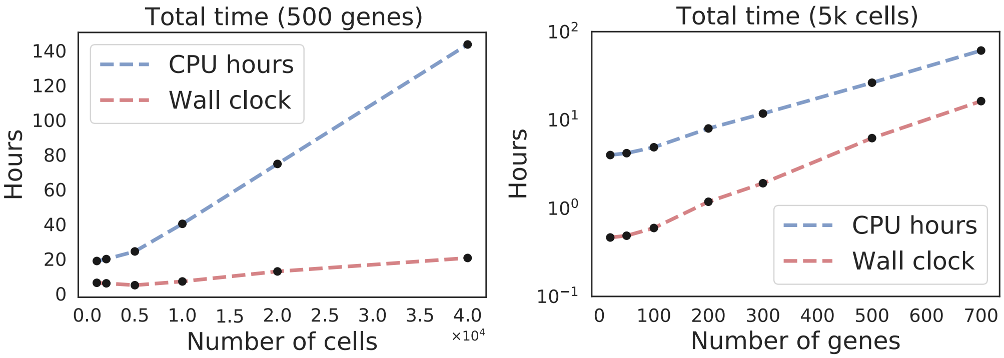

Appendix Figure S4: **Runtime of Stator's nextflow pipeline for increasing numbers of cells (left) or genes (right), using a fixed set of HPC resource**. Runtime is linear in the number of cells and exponential in the number of genes. The 'wall clock' time in red shows the total amount of time lapsed between the beginning and end of the nextflow process, including the scheduling time on the HPC, and corresponds to the real time spent to obtain results. CPU time includes only the hours taken for tasks spent on the CPU. However, due to parallelisation, the CPU time will be longer than the wall clock. The wall clock depends on availability of HPC parallelisation resources. For example, for the 10X data set with 19,000 cells and 1,000 genes, the run time was approximately 5 days on the Edinburgh University High Performance Compute Cluster (Eddie), parallelised over multiple cores corresponding to 2,500 CPU hours, as reported by the Nextflow pipeline manager.

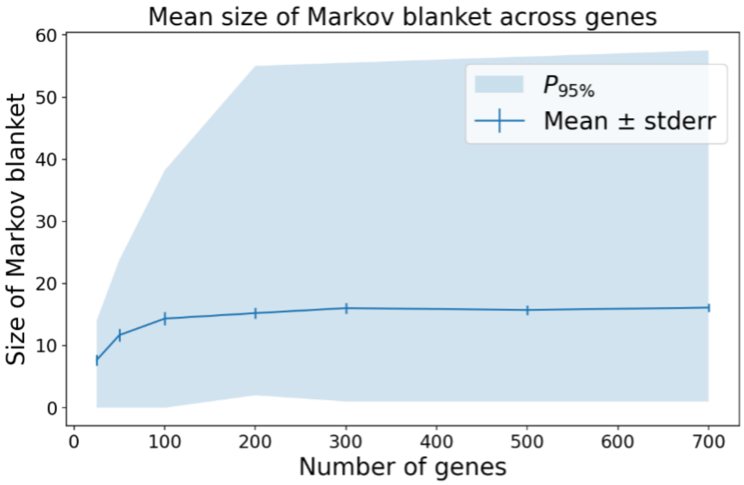

Appendix Figure S5: The mean size of the Markov blanket stabilises to within the standard error on the mean when calculations include at least 200 most highly variable genes, as does the 95th percentile around the median. This plot was generated using neurons from the mouse brain dataset.

A.4. **Comparison with multimodal data.** We projected Stator states from the original merged RP and neuron dataset into a second dataset's 5,000 cells (3,343 cells after quality control) using only their scRNA-seq data (10XGenomics, 2021). This yielded states that were predicted by projection rather than being predicted by re-running Stator. Using single cell chromatin accessibility data from these same 3,343 cells we then showed that differential mRNA expression and differential chromatin accessibility between two states can occur for the same genes (Appendix Figure S10). This showed:

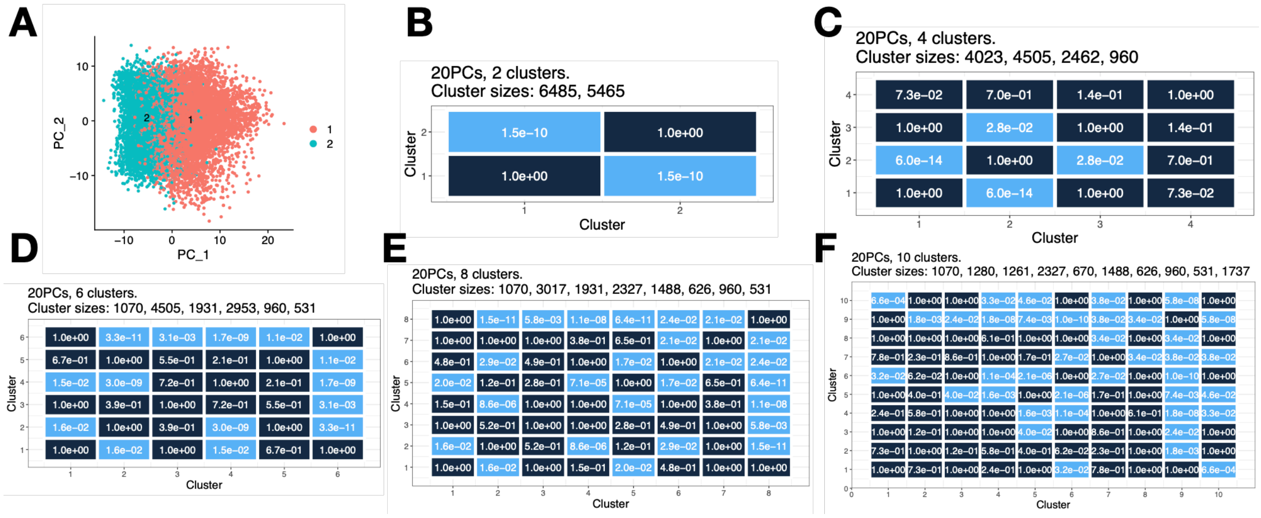

Appendix Figure S6: **Embryonic radial glial cell precursor cell hierarchical clustering analysis, with p-value quantification bounding for selective inference (Gao et al., 2022).** We applied standard single cell clustering to the non-binarised data, followed by pair-wise p-value quantification of cluster significance (Gao et al., 2022). In panels B-F, light blue represents pairwise significantly distinct clusters with Bonferroni corrected p-value < 0.05, whereas dark blue represents not significantly distinct clusters. **(A)** According to the clustering analysis this dataset is relatively homogeneous, containing two significantly distinct clusters, shown in a PCA plot. **(B)** Heatmap showing the p-value between clusters when choosing $k = 2$ clusters. **(C-D)** Heatmaps showing statistical support for clusters when choosing $k = 4, 6, 8, 10$ (C, D, E, F). Under these scenarios, not every cluster pair is significantly separated ($p < 0.05$ after correction with the Bonferroni method). To declare the final number of distinct clusters, we take the largest number of clusters such that all clusters are pairwise significantly distinct as k changes. In this case, it can be observed from panels B and C, that clusters #1, 2 are significantly pairwise distinct, but clusters #3, 4 are not. Therefore, we declare $k = 2$ distinct clusters.

(1) that the transcriptomic heterogeneity identified in the first dataset (RPs and neurons) is evident also in the second dataset, and (2) that this heterogeneity is evident concordantly in both altered gene expression and open chromatin.

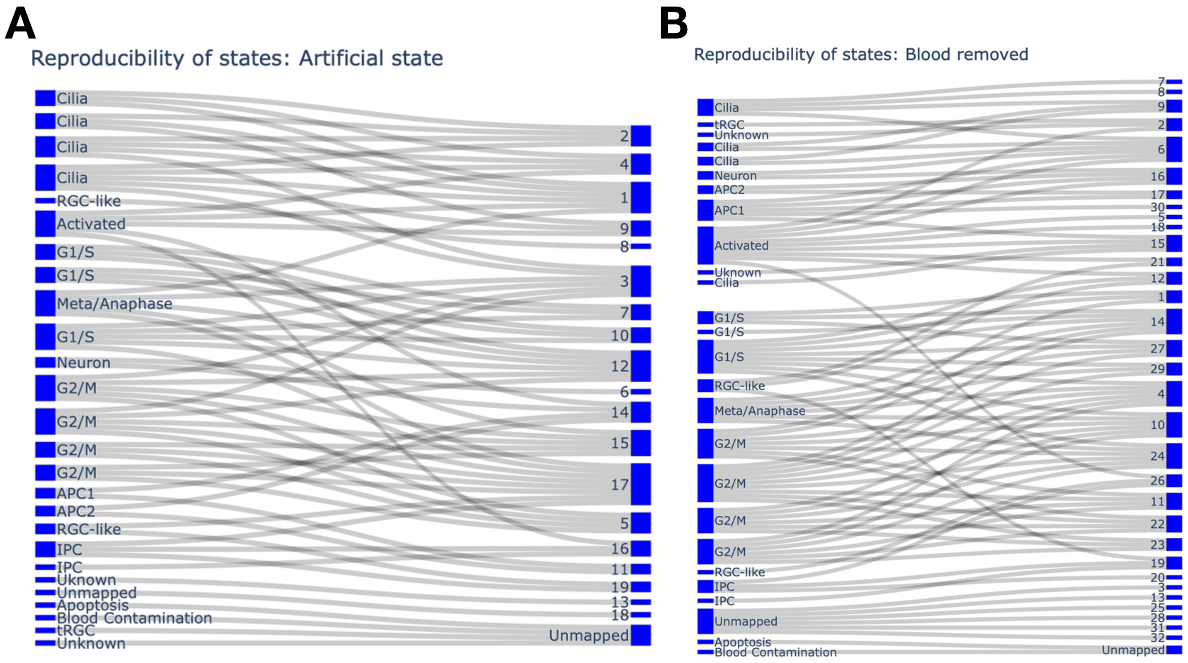

Appendix Figure S7: Simulations indicating an artificially induced d-tuple (panel A) and an artificially removed state (blood contamination, panel B), are recovered and removed by Stator, respectively. In each panel the left-hand blue boxes represent the original states, and the right-hand blue boxes represent the new Stator run. The lines map the states as described in the text. The lines map to multiple states, as expected, since Stator allows cell to exist in multiple states. **(A)** A fictitious d-tuple of genes is induced by randomly choosing 90% of the cells where (Cited2, Basp1, Fhl1) $= (1, 0, 1)$ and changing this to $(1, 1, 1)$. Stator is rerun, and this duple is recovered as part of state 14, with FDR < 0.05 and $>$ 6-fold enrichment, at the default Dice dissimilarity threshold. **(B)** Cells tagged as blood contamination in the original run were removed and Stator was re-run. The results indicate that the blood contamination state (bottom row) does not map to any state in the new run, as expected.

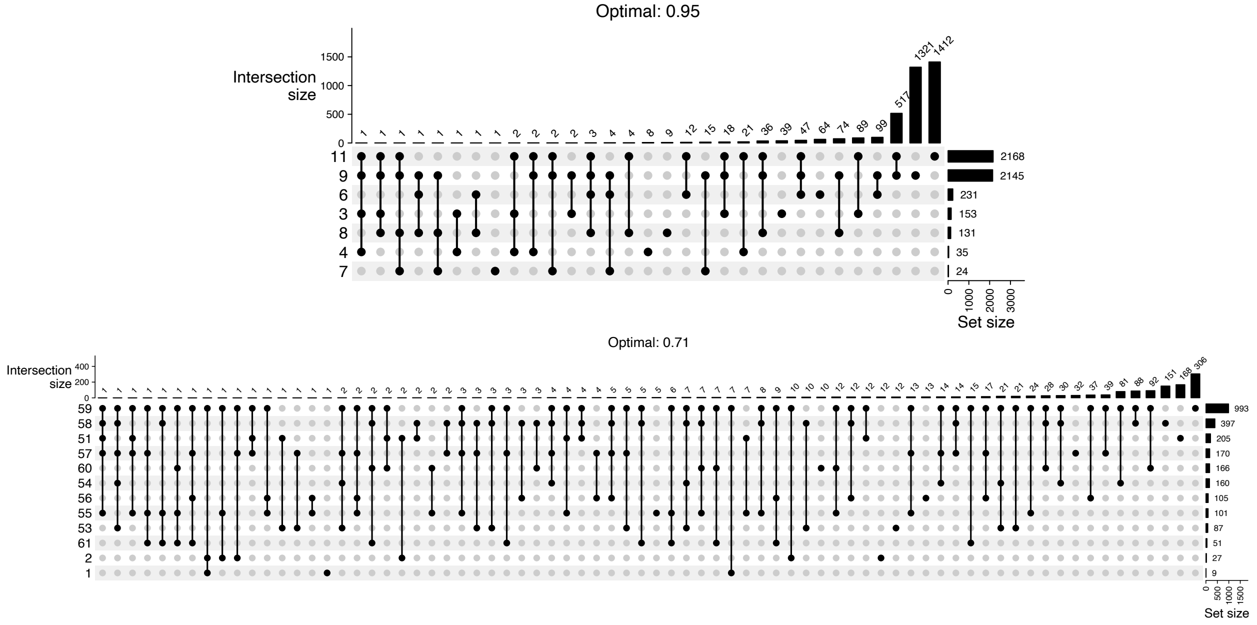

Appendix Figure S8: Upset plot of the number of co-labelled cells amongst cell-cycle states, presented in Figure 2B (top) and Figure EV3 (bottom).

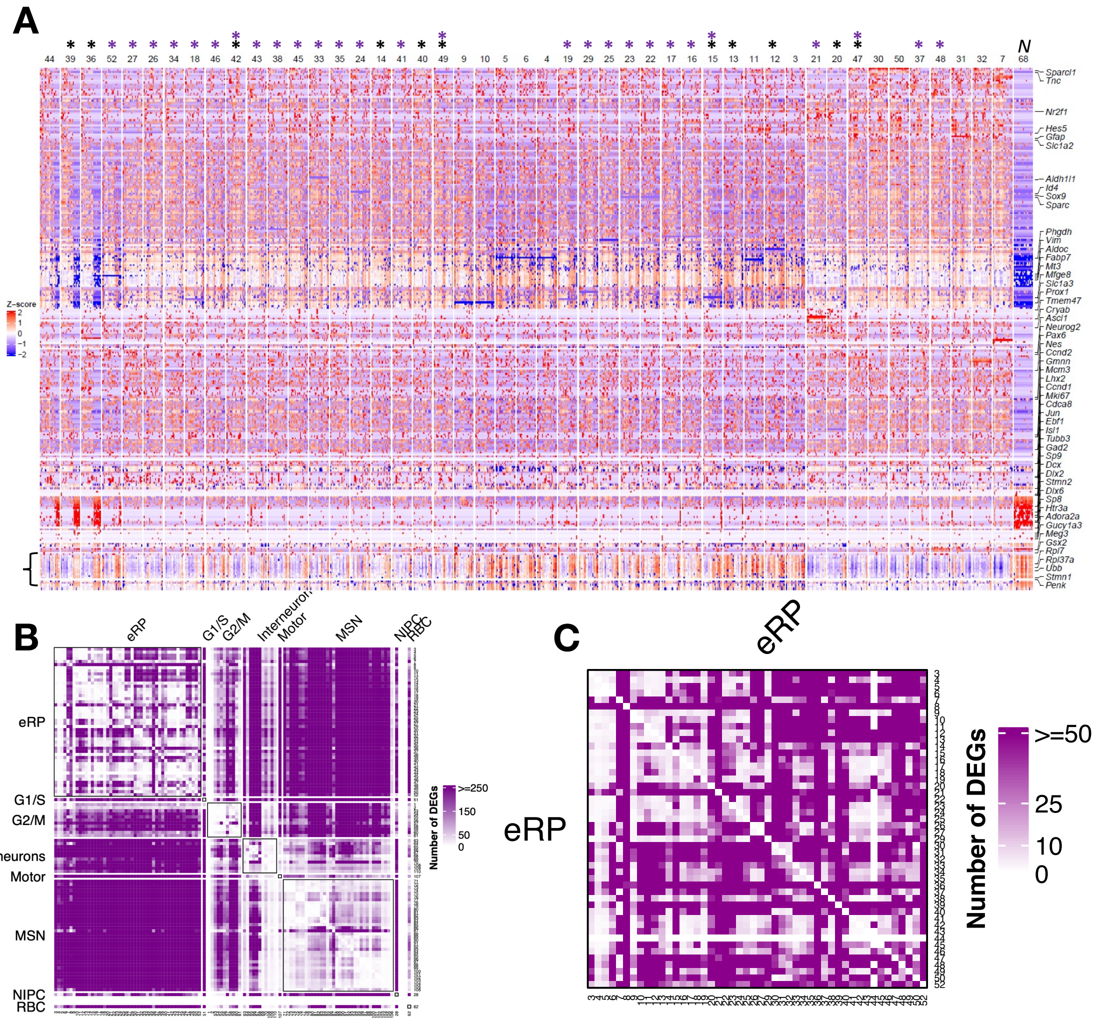

Appendix Figure S9: **(A)** Heatmap of unbinarised single cell gene expression across 47 embryonic radial glial precursor cell (eRP) states and, for comparison, a single *Tubb3+* neuron state (State 68 ["N"]: *Slain1* [1], *Phgdh* [0], *Tubb3* [1]). Top: Asterisks indicate states with higher expression of neuronal marker s2s-DEGs than State 44 (black) or State 40 (purple). Numbers indicate States. Right: The 20 highest differentially expressed genes per state in s2oDEGs analysis, with, in addition, E17.5 eRP expressing genes listed in Supplementary Table S3 of Yuzwa et al. (2017) are shown. Horizontal lines reflect genes that are not expressed (blue) or always expressed (red) in a State's cells. Heterogeneous gene expression across states is evident, for example, due to high expression of ribosomal protein and other genes (indicated by a bracket, lower left), an indicator of activated neural stem cells (Borrett et al., 2022; Dulken et al., 2017). **(B)** Heatmap of numbers of s2s-DEGs for the merged RP and neuron dataset. Colour indicates the number of DEGs. Stator state pairs with different labels have more s2s-DEGs than those with the same label, as expected. **(C)** As in (B), but for embryonic RP states only. This shows considerable transcriptional heterogeneity among these Stator RP states.

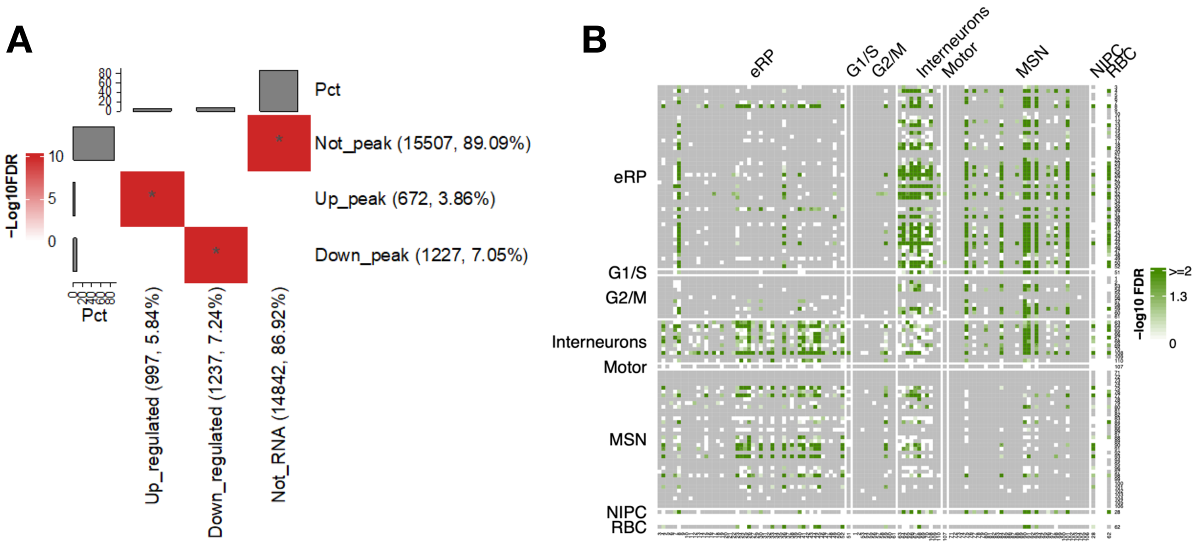

Appendix Figure S10: **(A)** Differential mRNA expression and differential chromatin accessibility between two states tend to occur for the same genes in the same single cells. Stator states #93 (labelled as neurons) and #44 (RPs), from the merged RP and neuron dataset, were projected into a multi-omic (scRNA-Seq and scATAC-Seq) dataset from the same condition, E18 mouse brain. X-axis: Up- or down-regulated s2s-DEGs between cells labelled by projection as state #93 over state #44 (as an illustrative example) or non-expressed genes. Y-axis: Genes' ATAC-seq peaks that are higher or lower between these two states' cells, or else unchanged. The heatmap indicates the enrichment of genes that are both differentially expressed between these states' cells and contain differentially open chromatin between these same cells. Differential peaks were extracted via the Seurat function `FindMarkers`, then the closest genes to the genomic regions of these differential peaks were extracted using the `ClosestFeature` function. For FDR control, the BH procedure was applied (Benjamini and Hochberg, 1995). Values in parentheses represent numbers and percentages of genes (X-axis) and peaks (Y-axis), respectively. **(B)** In green: Pairs of projected states that show significant enrichment of genes that are both up-regulated in expression and have increased chromatin accessibility in the same cell. Rather than a single pair of states (as in (A)), the enrichment of up-regulated expression and increased chromatin accessibility was tested for all pairs of states in cells labelled by projection from the merged RP and neuron dataset into the multi-omic dataset. Concomitant gene up-regulation and increased chromatin expression occurs more frequently between pairs of interneuron projected states, than between embryonic RP states or between medium spiny neuron (MSN) states, indicating that there is greater molecular diversity among these interneuron states. The original merged (neuron and RP) dataset and the multiomic data set may not capture molecular heterogeneity equally. Similarly, the multiomic dataset may not capture expression and chromatin heterogeneity equally.

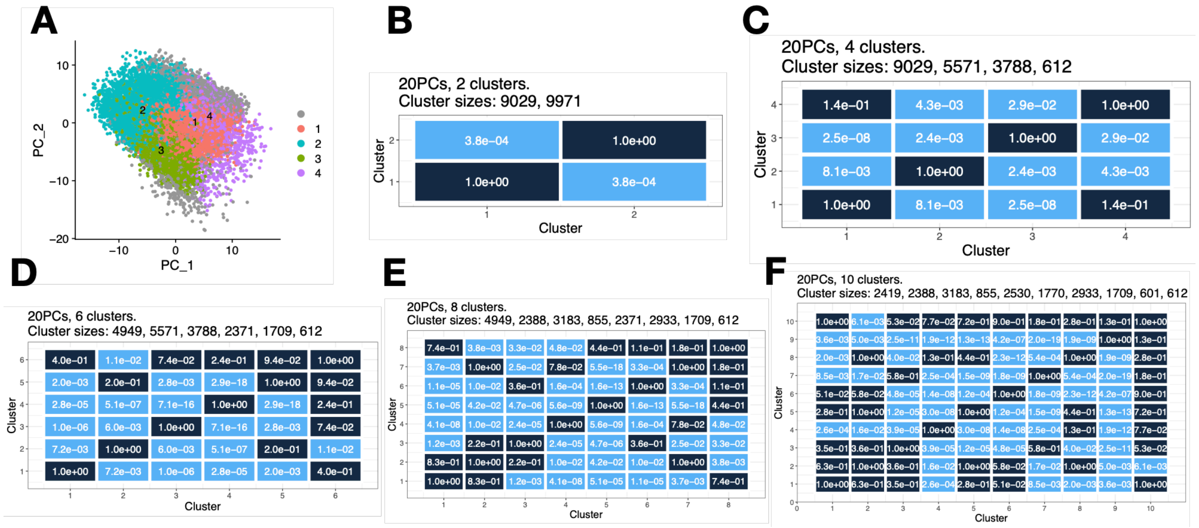

Appendix Figure S11: **Hierarchical clustering of E18 developmental neurons, with p-value quantification bounding for selective inference (Gao et al., 2022)**. We applied standard single cell clustering to the non-binarised data, followed by pair-wise p-value quantification of cluster significance (Gao et al., 2022). In panels B-F, light blue represents pairwise significantly distinct clusters with Bonferroni correction p-value < 0.05, whereas dark blue represents not significantly distinct clusters. **(A)** PCA plot showing clustering of cells. According to this clustering analysis the dataset contains two significantly distinct clusters. **(B)** Heatmap showing the p-value for $k = 2$ clusters. It leads to significant differences between the clusters. **(C-F)** Heatmaps showing p-values between cluster pairs for $k = 4, 6, 8, 10$. Under these scenarios, not every cluster pair is significantly separated ($p < 0.05$ after correction with the Bonferroni method). To declare the final number of distinct clusters, we take the largest number of clusters such that all clusters are pairwise significantly distinct as k changes. In this case, it can be observed from panel D that clusters $\#1, 2, 3, 4$ are significantly pairwise distinct, but not clusters $\#5, 6$. Therefore, we declare $k = 4$ distinct clusters.

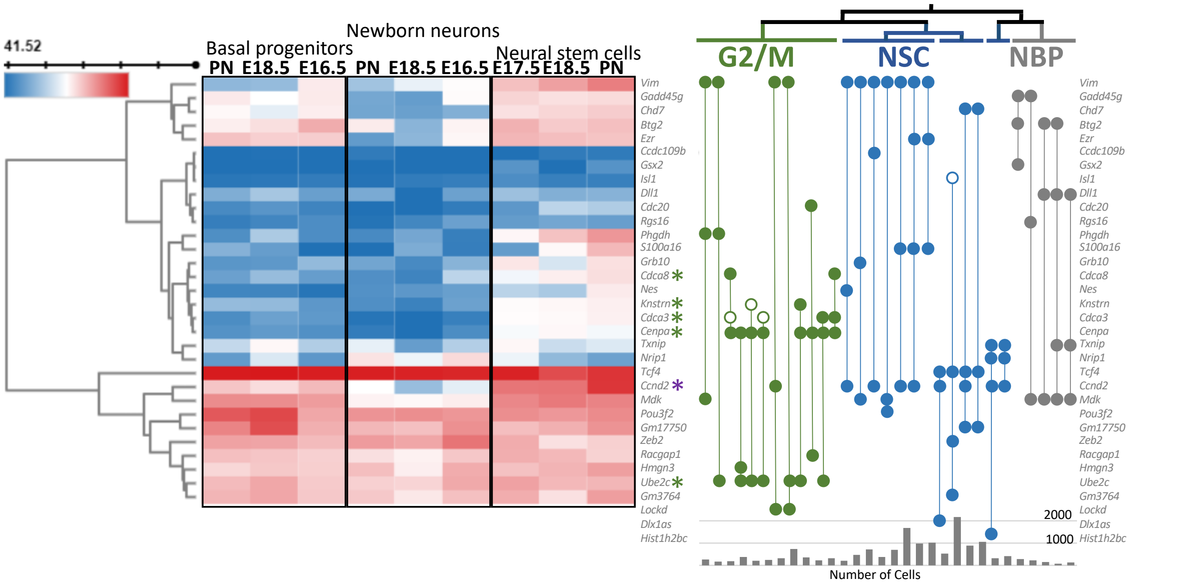

Appendix Figure S12: **Example of a Stator state (#26), identified using a high Dice dissimilarity (0.94), which can be further separated into biological states, here G2/M cell cycle phase, neural stem cells (NSC) and newborn progenitor (NPC) cells**. Differential expression across embryonic (E) day 16.5 to postnatal day 1 of genes specifying d-tuples in neuron developmental data set #26. The heatmap indicates gene expression in NSCs, basal progenitor cells and newborn neurons (NBN) taken from NeuroStemX, an external data set https://neurostemx.ethz.ch (Mukhtar et al., 2022); expression data for *Lockd*, *Dlx1as* and *Hist1h2bc* was not available. Based on literature markers for G2/M cell cycle phase (green asterisk), NSCs and NB progenitors (NBP; *Btg2* (Micheli et al., 2015); *Mdk* (Winkler and Yao, 2014)), #26 d-tuples were partitioned into G2/M, NSC and NB progenitor states (in green, blue and grey, respectively); tuples are indicated by vertical lines linking either three or four genes (filled circle signifies expressed, unfilled circle not expressed genes). High cyclin D2 (*Ccnd2*; purple asterisk) mRNA levels separates self-renewing cells from NBNs (Tsunekawa et al., 2014); *Ccnd2*-/- mice lack newly born neurons (Kowalczyk et al., 2004). Three tuples containing *Dll1* are notable in specifying newborn progenitor cells. *Dll1* is known to segregate asymmetrically during mitosis of NSCs, subsequently being inherited by differentiating cells (Kawaguchi et al., 2013).

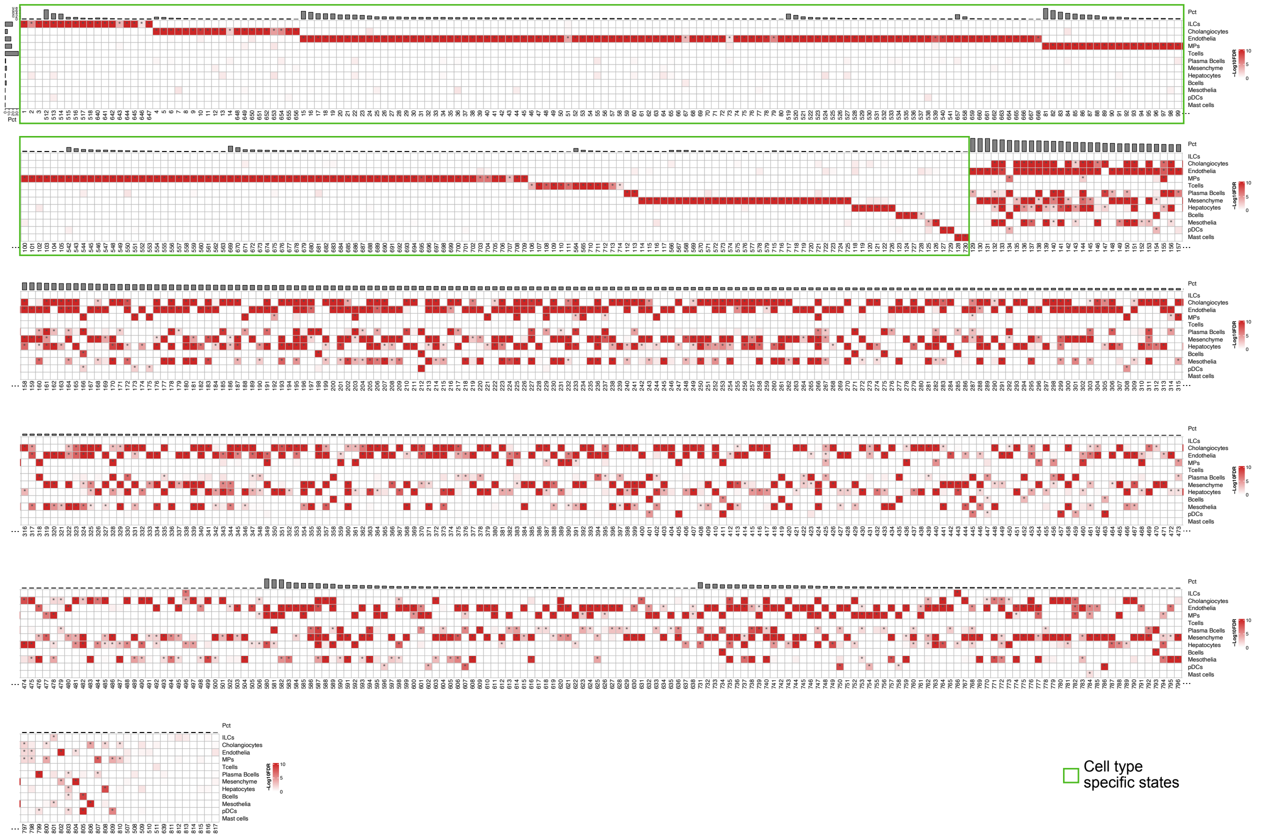

Appendix Figure S13: **Reducing the d-tuple enrichment threshold and Dice similarity increases the coverage of Stator states that can be labelled by external cell type annotations.** By simultaneously decreasing (i) the enrichment threshold used in Figure 5 from 8 to 2 (Log2FC=3 to Log2FC=1), and (ii) the Dice similarity threshold from 0.97 to 0.50, every external cell type annotation is enriched in one or more Stator state. States linked to only one cell type annotation are highlighted within green bordered boxes.
