# Supplementary material for: High order expression dependencies finely resolve cryptic states and subtypes in single cell data
Source: Mol Syst Biol. 2025 Jan 2;21(2):173–207. doi: 10.1038/s44320-024-00074-1 (PMC11790937; doi:10.1038/s44320-024-00074-1)
Supplement: Supplementary file 30 — Expanded View Figures [file 44320_2024_74_MOESM30_ESM.pdf]

Expanded View Figures

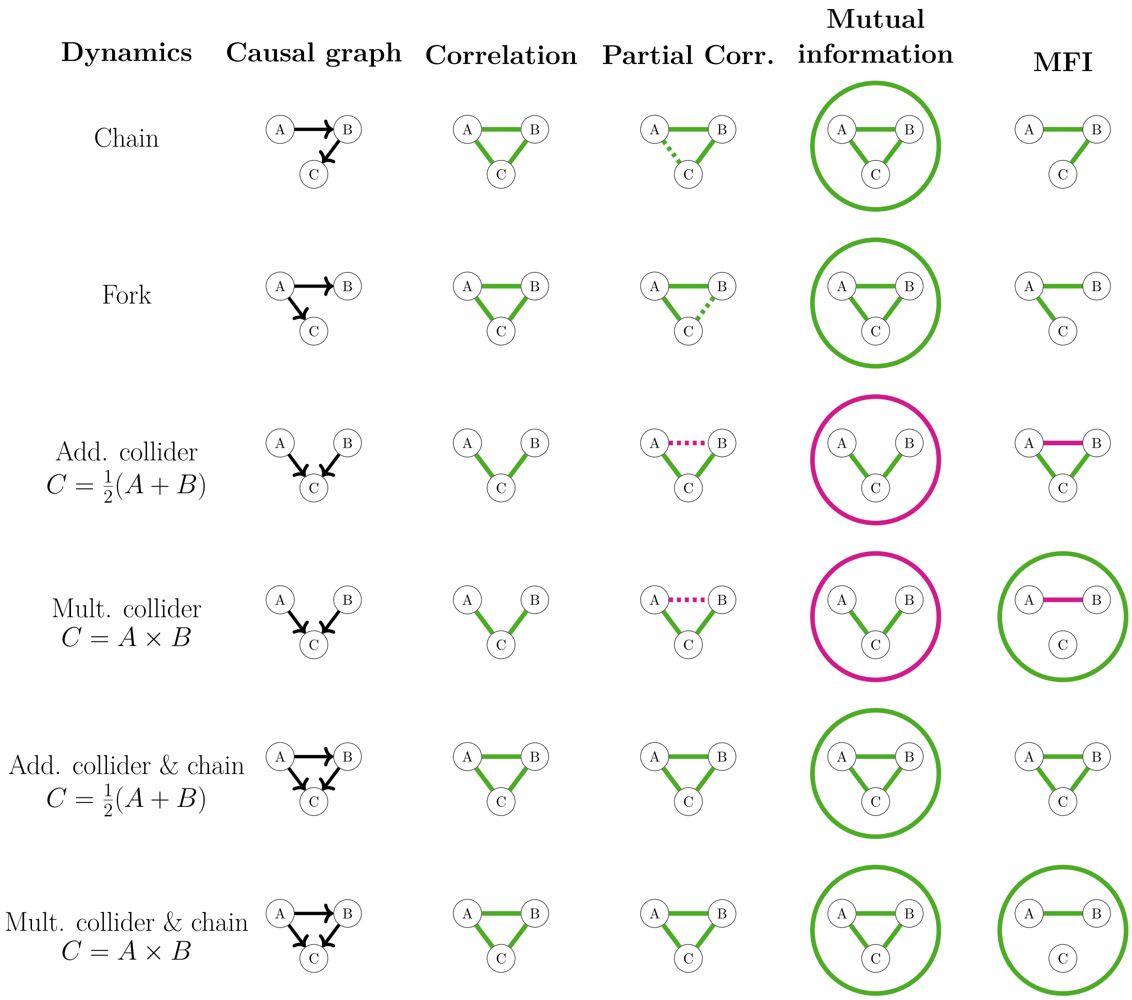

Figure EV1. Expanded view for Fig. 1.

Comparison of MFIs with other estimators of dependence. Different causal dynamics lead to different association metrics, and only MFIs can distinguish all 6 scenarios and reveal the combinatorial effect of a multiplicative interaction. Green edges denote positive values, red edges denote negative values, circles denote a 3-point quantity, and dashed lines show edges that show marginal significance that depends on the level of simulated noise. Correlations and mutual information cannot distinguish between most dynamics, and while partial correlation can, for certain noise levels, identify the correct pairwise relationships, it falls short of distinguishing additive from multiplicative dynamics. See Appendix Fig. S1 for the simulation parameters and precise values. Reproduced from (Jansma, 2023a) with permission from the author.

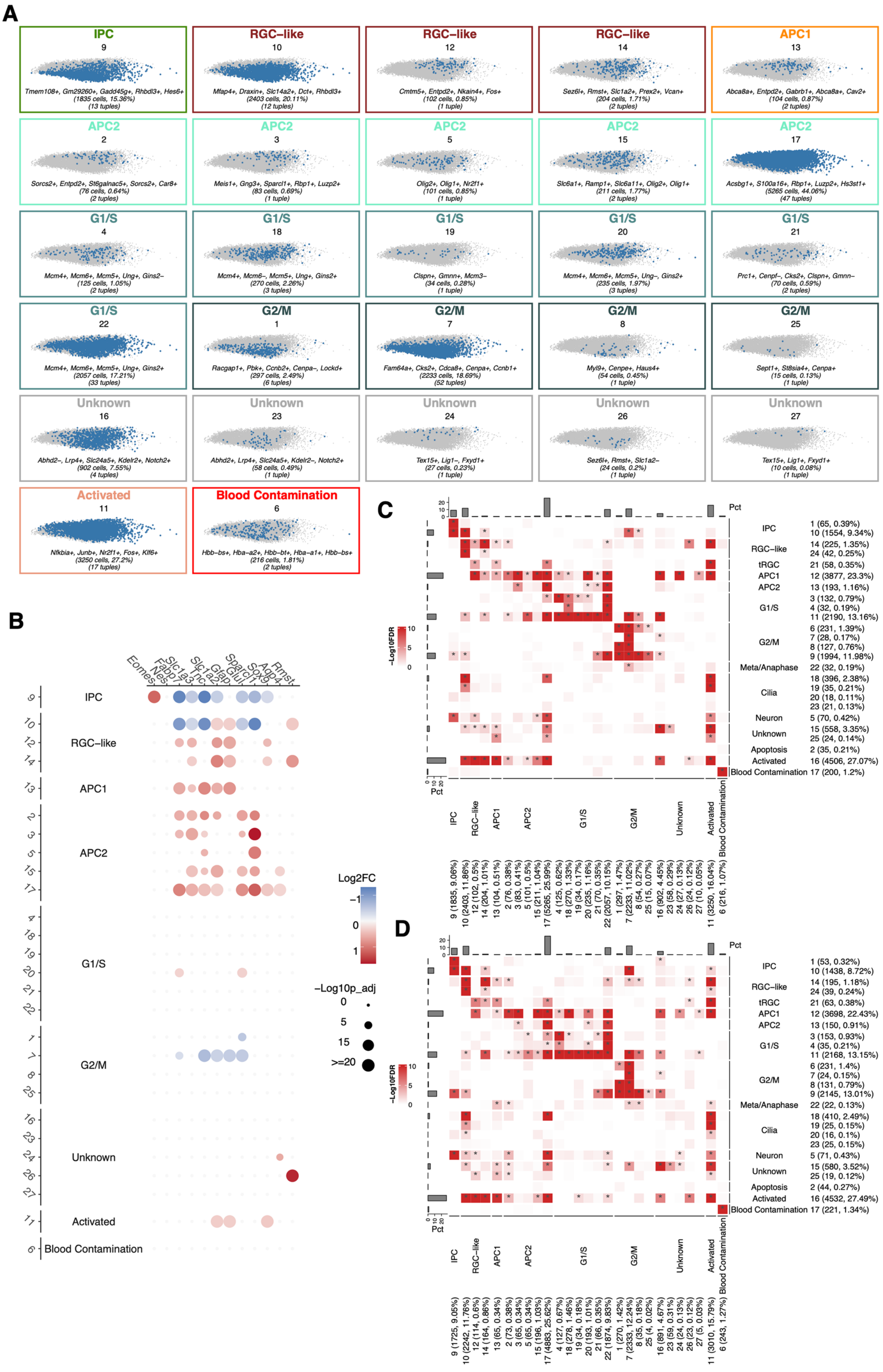
A
IPC
9
Tmem108+, Gm29260+, Gadd45g+, Rhbdl3+, Hes6+
(1835 cells, 15.36%)
(13 tuples)
RGC–like
10
Mfap4+, Draxin+, Slc14a2+, Dct+, Rhbdl3+
(2403 cells, 20.11%)
(12 tuples)
RGC–like
12
Cmtm5+, Entpd2+, Nkain4+, Fos+
(102 cells, 0.85%)
(1 tuple)
RGC–like
14
Sez6l+, Rmst+, Slc1a2+, Prex2+, Vcan+
(204 cells, 1.71%)
(2 tuples)
APC1
13
Abca8a+, Entpd2+, Gabrb1+, Abca8a+, Cav2+
(104 cells, 0.87%)
(2 tuples)
APC2
2
Sorcs2+, Entpd2+, St6galnac5+, Sorcs2+, Car8+
(76 cells, 0.64%)
(2 tuples)
APC2
3
Meis1+, Gng3+, Sparcl1+, Rbp1+, Luzp2+
(83 cells, 0.69%)
(1 tuple)
APC2
5
Olig2+, Olig1+, Nr2f1+
(101 cells, 0.85%)
(1 tuple)
APC2
15
Slc6a1+, Ramp1+, Slc6a11+, Olig2+, Olig1+
(211 cells, 1.77%)
(2 tuples)
APC2
17
Acsbg1+, S100a16+, Rbp1+, Luzp2+, Hs3st1+
(5265 cells, 44.06%)
(47 tuples)
G1/S
4
Mcm4+, Mcm6+, Mcm5+, Ung+, Gins2–
(125 cells, 1.05%)
(2 tuples)
G1/S
18
Mcm4+, Mcm6–, Mcm5+, Ung+, Gins2+
(270 cells, 2.26%)
(3 tuples)
G1/S
19
Clspn+, Gmnn+, Mcm3–
(34 cells, 0.28%)
(1 tuple)
G1/S
20
Mcm4+, Mcm6+, Mcm5+, Ung–, Gins2+
(235 cells, 1.97%)
(3 tuples)
G1/S
21
Prc1+, Cenpf–, Cks2+, Clspn+, Gmnn–
(70 cells, 0.59%)
(2 tuples)
G1/S
22
Mcm4+, Mcm6+, Mcm5+, Ung+, Gins2+
(2057 cells, 17.21%)
(33 tuples)
G2/M
1
Racgap1+, Pbk+, Ccnb2+, Cenpa–, Lockd+
(297 cells, 2.49%)
(6 tuples)
G2/M
7
Fam64a+, Cks2+, Cdca8+, Cenpa+, Ccnb1+
(2233 cells, 18.69%)
(52 tuples)
G2/M
8
Myl9+, Cenpe+, Haus4+
(54 cells, 0.45%)
(1 tuple)
G2/M
25
Sept1+, St8sia4+, Cenpa+
(15 cells, 0.13%)
(1 tuple)
Unknown
16
Abhd2–, Lrp4+, Slc24a5+, Kdelr2+, Notch2+
(902 cells, 7.55%)
(4 tuples)
Unknown
23
Abhd2+, Lrp4+, Slc24a5+, Kdelr2–, Notch2+
(58 cells, 0.49%)
(1 tuple)
Unknown
24
Tex15+, Lig1–, Fxyd1+
(27 cells, 0.23%)
(1 tuple)
Unknown
26
Sez6l+, Rmst+, Slc1a2–
(24 cells, 0.2%)
(1 tuple)
Unknown
27
Tex15+, Lig1+, Fxyd1+
(10 cells, 0.08%)
(1 tuple)
Activated
11
Nfkbia+, Junb+, Nr2f1+, Fos+, Klf6+
(3250 cells, 27.2%)
(17 tuples)
Blood Contamination
6
Hbb–bs+, Hba–a2+, Hbb–bt+, Hba–a1+, Hbb–bs+
(216 cells, 1.81%)
(2 tuples)
B
Eomes
Fabp7
Nes
Slc1a3
Tnc
Slc1a2
Gfap
Glul
Sparcl1
Sox9
Aqp4
Rmst
IPC
RGC–like
APC1
APC2
G1/S
G2/M
Unknown
Activated
Blood Contamination
Log2FC
–Log10p_adj
>=20
C
D
Pct
–Log10FDR
IPC
RGC–like
tRGC
APC1
APC2
G1/S
G2/M
Meta/Anaphase
Cilia
Neuron
Unknown
Apoptosis
Activated
Blood Contamination

 © The Author(s)

Figure EV2. Expanded view for Fig. 2.

Disjoint RP dataset tested for reproducibility. (**A**) Stator identifies 27 states for a disjoint set of $N = 11,950$ embryonic radial glial precursor-like cells (set RP2) at maximum modularity, annotated on the basis of d-tuple and s2o-DE genes as markers for cell types or cell cycle phases. (**B**) Dot plot illustrating differential expression of astrocytogenesis marker genes across all 27 Stator states. The size of the dots represents the -$\log_{10}$(Seurat p-val-adj) from differential gene expression testing between a state and all other states. Colour intensity reflects the $\log_2$(FC) of gene expression. (**C**) The *x* axis shows the states obtained by running Stator on the RP2 dataset, while the *y* axis shows states obtained by running Stator on the dataset from Fig. 2 (set RP1). The RP1 states, obtained by running Stator on RP1 cells, are projected onto RP2 cells. The enrichment of these projected RP1 states in RP2 Stator states is computed using a hypergeometric test followed by the BH procedure to control the FDR at 5%. (**D**) As (**C**), but for RP1 cells. The *y* axis shows the RP1 states, while the *x* axis shows states obtained by running Stator on RP2 cells. The RP2 states are projected onto RP1 cells, and their enrichment in each RP1 cells' state is computed using a hypergeometric test followed by the BH procedure to control the FDR at 5%. These panels demonstrate the reproducibility of Stator states on two disjoint sets of cells in the same biological condition.

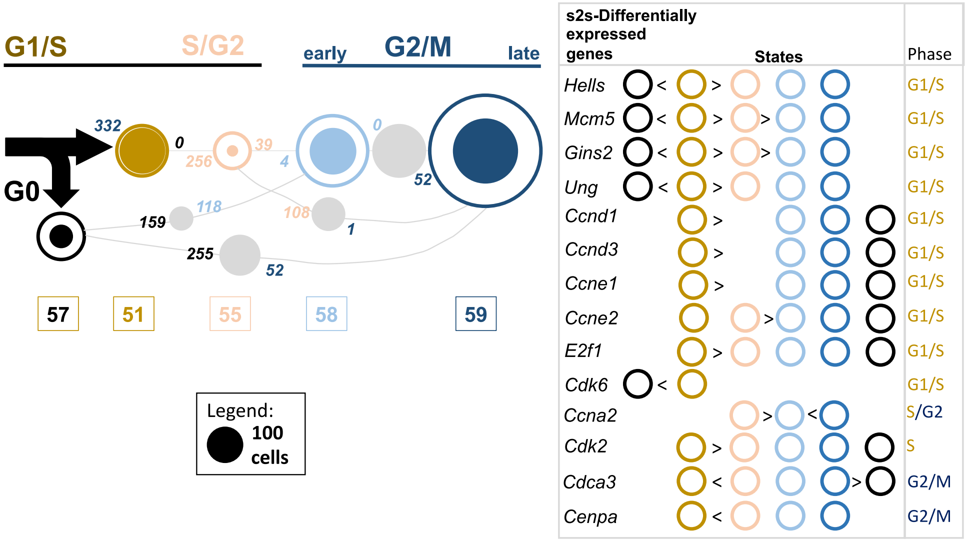

Figure EV3. Expanded view for Fig. 3.

s2s-DEG analysis for 5 cell cycle states. Numbers of cells labelled with any one of 5 cell cycle states (#57, 51, 55, 58, and 59) in embryonic RPs and neurons; areas of circles are proportional to their number (see legend). Filled circles indicate numbers of cells labelled with only of these single cell cycle states. Grey circles' areas indicate numbers of cells labelled with two cell cycle states, those indicated by lines. Numbers of significantly differentially expressed genes between cell cycle state pairs (i.e., s2s-DEGs) are provided between the two states being compared; their colours refer to the state showing higher expression. For clarity, state pairs with ≥ 25 cells are shown. DEGs between any two states, including state pairs with fewer than 25 co-labelled cells, are provided in Dataset EV12. Appendix Fig. S8B additionally provides the number of co-labelled cells between any two states. Right: s2s-DEGs are indicated by ">" or "<" symbols; for example, *Hells* mRNA expression is significantly higher in State #51 over States #57, 55, 58 and 59. Early/late G1/S or G2/M cell cycle phase labels (top) were assigned using these mRNAs' cell cycle phases known from high-throughput (top right; (Giotti et al, 2018)) and targeted experiments (*Ung* mRNA in late G1/S (Slupphaug et al, 1991) and *Cenpa* in G2 (Shelby et al, 1997)).

 © The Author(s)

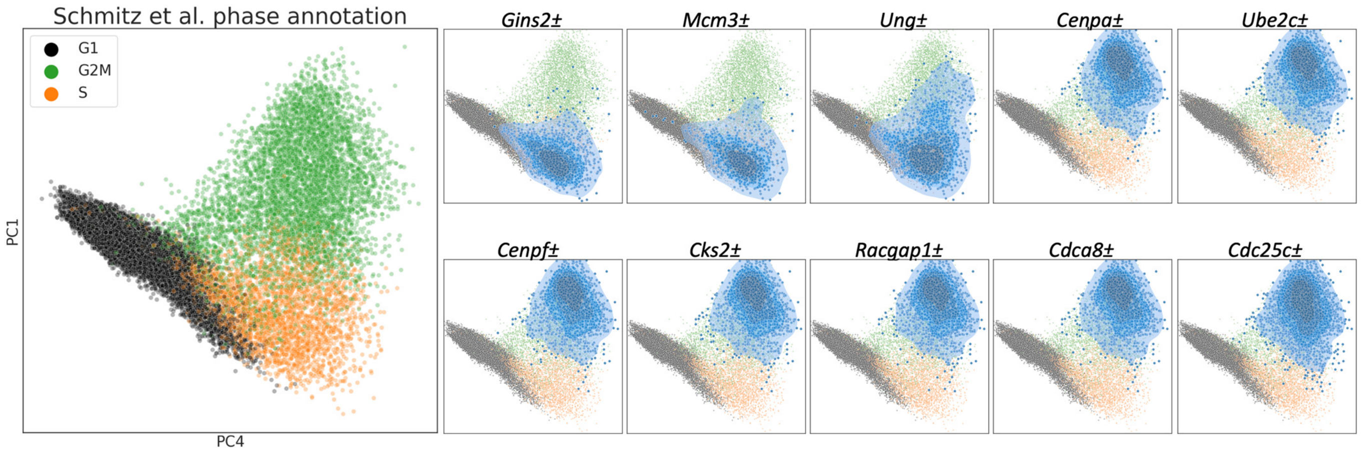

Figure EV4. Expanded view for Fig. 3.

Minus gene expression is required to specify cell cycle sub-phases. Left: External cell cycle annotations of a merged mouse brain dataset sourced from five different experiments (Schmitz et al, 2022), with the cells from dataset GSE93421 removed. Right: In contrast to Fig. 3B, highlighting cells (in blue) based only on the expression of all but one of the marker genes, leaving the gene indicated above each plot (the 'minus' gene) unrestricted, results in more diffuse cell cycle specificity, and simply marks cells in G1/S or G2/M phases.

© The Author(s)

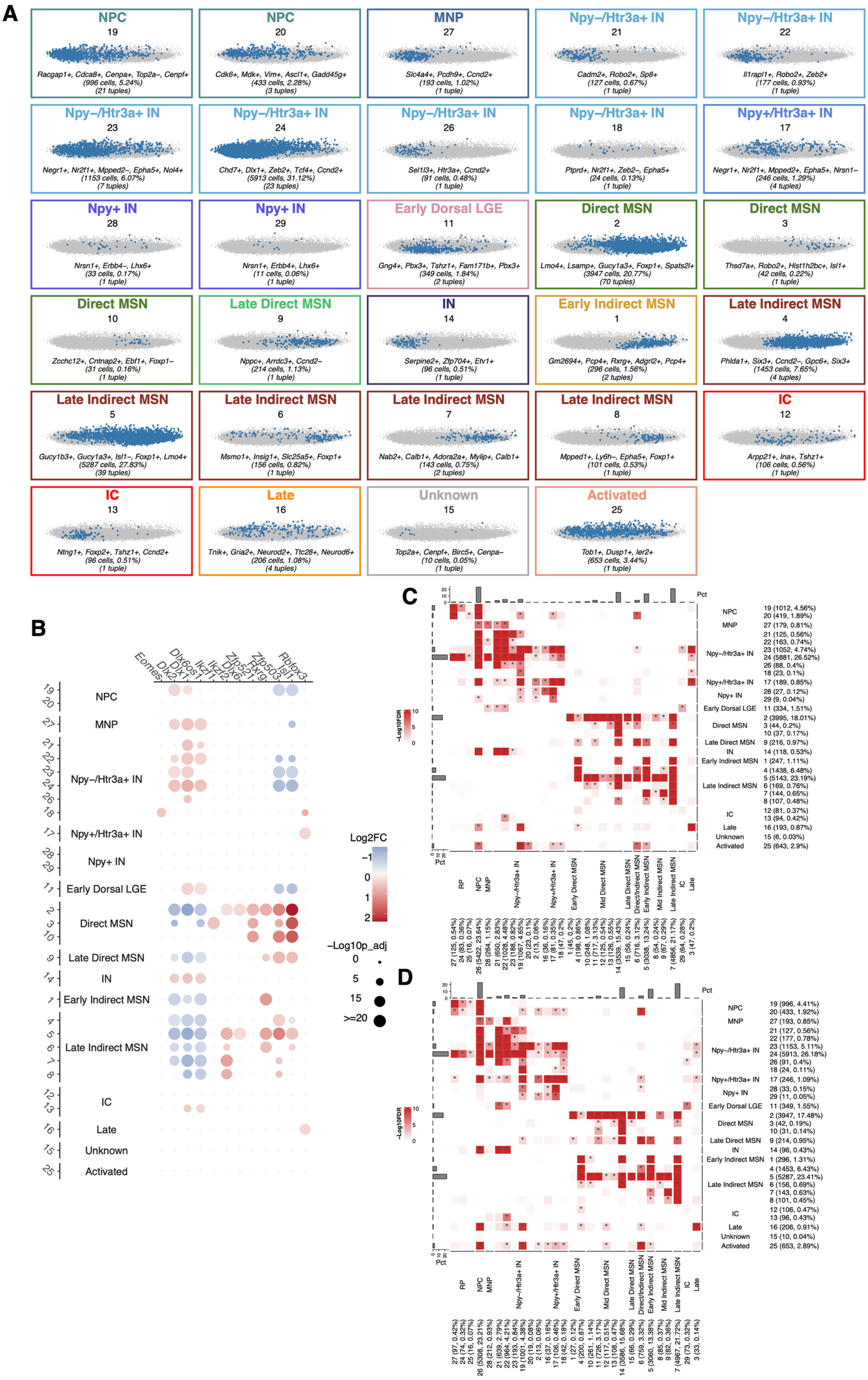
A
NPC
19
Racgap1+, Cdca8+, Cenpa+, Top2a–, Cenpf+
(996 cells, 5.24%)
(21 tuples)
NPC
20
Cdk6+, Mdk+, Vim+, Ascl1+, Gadd45g+
(433 cells, 2.28%)
(3 tuples)
MNP
27
Slc4a4+, Pcdh9+, Ccnd2+
(193 cells, 1.02%)
(1 tuple)
Npy–/Htr3a+ IN
21
Cadm2+, Robo2+, Sp8+
(127 cells, 0.67%)
(1 tuple)
Npy–/Htr3a+ IN
22
Il1rapl1+, Robo2+, Zeb2+
(177 cells, 0.93%)
(1 tuple)
Npy–/Htr3a+ IN
23
Negr1+, Nr2f1+, Mpped2–, Epha5+, Nol4+
(1153 cells, 6.07%)
(7 tuples)
Npy–/Htr3a+ IN
24
Chd7+, Dlx1+, Zeb2+, Tcf4+, Ccnd2+
(5913 cells, 31.12%)
(23 tuples)
Npy–/Htr3a+ IN
26
Sel1l3+, Htr3a+, Ccnd2+
(91 cells, 0.48%)
(1 tuple)
Npy–/Htr3a+ IN
18
Ptprd+, Nr2f1+, Zeb2–, Epha5+
(24 cells, 0.13%)
(1 tuple)
Npy+/Htr3a+ IN
17
Negr1+, Nr2f1+, Mpped2+, Epha5+, Nrsn1–
(246 cells, 1.29%)
(4 tuples)
Npy+ IN
28
Nrsn1+, Erbb4–, Lhx6+
(33 cells, 0.17%)
(1 tuple)
Npy+ IN
29
Nrsn1+, Erbb4+, Lhx6+
(11 cells, 0.06%)
(1 tuple)
Early Dorsal LGE
11
Gng4+, Pbx3+, Tshz1+, Fam171b+, Pbx3+
(349 cells, 1.84%)
(2 tuples)
Direct MSN
2
Lmo4+, Lsamp+, Gucy1a3+, Foxp1+, Spats2l+
(3947 cells, 20.77%)
(70 tuples)
Direct MSN
3
Thsd7a+, Robo2+, Hist1h2bc+, Isl1+
(42 cells, 0.22%)
(1 tuple)
Direct MSN
10
Zcchc12+, Cntnap2+, Ebf1+, Foxp1–
(31 cells, 0.16%)
(1 tuple)
Late Direct MSN
9
Nppc+, Arrdc3+, Ccnd2–
(214 cells, 1.13%)
(1 tuple)
IN
14
Serpine2+, Zfp704+, Etv1+
(96 cells, 0.51%)
(1 tuple)
Early Indirect MSN
1
Gm2694+, Pcp4+, Rxrg+, Adgrl2+, Pcp4+
(296 cells, 1.56%)
(2 tuples)
Late Indirect MSN
4
Phlda1+, Six3+, Ccnd2–, Gpc6+, Six3+
(1453 cells, 7.65%)
(4 tuples)
Late Indirect MSN
5
Gucy1b3+, Gucy1a3+, Isl1–, Foxp1+, Lmo4+
(5287 cells, 27.83%)
(39 tuples)
Late Indirect MSN
6
Msmo1+, Insig1+, Slc25a5+, Foxp1+
(156 cells, 0.82%)
(1 tuple)
Late Indirect MSN
7
Nab2+, Calb1+, Adora2a+, Mylip+, Calb1+
(143 cells, 0.75%)
(2 tuples)
Late Indirect MSN
8
Mpped1+, Ly6h–, Epha5+, Foxp1+
(101 cells, 0.53%)
(1 tuple)
IC
12
Arpp21+, Ina+, Tshz1+
(106 cells, 0.56%)
(1 tuple)
IC
13
Ntng1+, Foxp2+, Tshz1+, Ccnd2+
(96 cells, 0.51%)
(1 tuple)
Late
16
Tnik+, Gria2+, Neurod2+, Ttc28+, Neurod6+
(206 cells, 1.08%)
(4 tuples)
Unknown
15
Top2a+, Cenpf+, Birc5+, Cenpa–
(10 cells, 0.05%)
(1 tuple)
Activated
25
Tob1+, Dusp1+, Ier2+
(653 cells, 3.44%)
(1 tuple)
B
NPC
MNP
Npy–/Htr3a+ IN
Npy+/Htr3a+ IN
Npy+ IN
Early Dorsal LGE
Direct MSN
Late Direct MSN
IN
Early Indirect MSN
Late Indirect MSN
IC
Late
Unknown
Activated
Log2FC
–Log10p_adj
0
5
15
>=20
C
Pct
–Log10FDR
D

Figure EV5. Expanded view for Fig. 4.

A second dataset of developmental neurons testing for reproducibility. Disjoint neurons dataset for reproducibility. (**A**) Stator identifies 29 states for a disjoint set of $N = 19,000$ developmental neurons (set N2) at a Dice dissimilarity of 0.94, annotated when d-tuple and/or s2o-DEGs are marker genes for cell (sub)types or states. (**B**) Dot plot illustrating differential expression of neurogenesis marker genes across all Stator states. The size of the dots represents the -$\log_{10}$(Seurat p-val-adj) from differential expression testing between a state and all other states. Colour intensity represents the $\log_2$(FC) of gene expression. (**C**) The *x* axis shows the states obtained by running Stator on the Fig. 4 dataset (set N1), while the y axis shows states obtained by running Stator on the dataset in panel A (set N2). The N1 states, obtained by running Stator on N1 cells, are projected onto N2 cells. The enrichment of these projected states in N2 Stator states is computed using a hypergeometric test followed by the BH procedure to control the FDR at 5%. (**D**) As (**C**), but for N1 cells. The y axis shows the N2 states, while the x axis shows states obtained by running Stator on N1 cells. The N2 states are projected onto N1 cells, and their enrichment in each N1 cells' state is computed using a hypergeometric test followed by the BH procedure to control the FDR at 5%. These panels demonstrate reproducibility of Stator states on two disjoint sets of cells in the same biological condition.

© The Author(s)

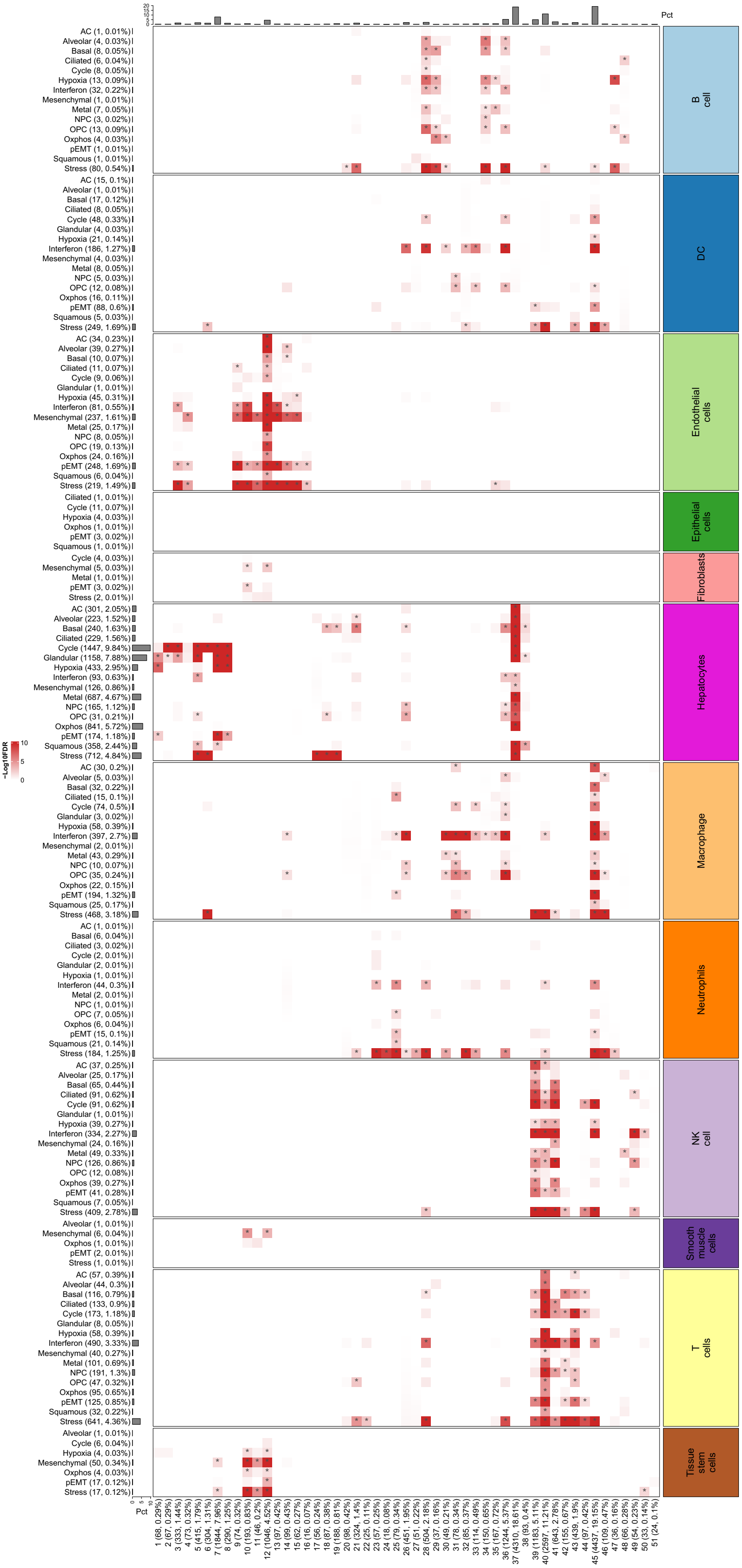

© The Author(s)

Figure EV6. Expanded view for Fig. 6.

Stator states (columns) that are significantly enriched in HCC cells previously doubly-annotated by cell type (right) and cell state (left) inferred using singleR and NMF, respectively.

© The Author(s)
